# The role of the Revised Trauma Score in penetrating trauma

**DOI:** 10.1590/0100-6991e-2026005925-en

**Published:** 2026-04-09

**Authors:** STEFFANY BARBOSA REIS, MATHEUS PORTUGAL, MARIA TAVARES, MALU ADAN, GABRIEL SANTANA, ANA ROMEO

**Affiliations:** 1 - Hospital do Subúrbio Salvador - Salvador - BA - Brasil

**Keywords:** Predictive Value of Tests, Wounds, Penetrating, Trauma Severity Indices, Valor Preditivo dos Testes, Ferimentos Penetrantes, Índices de Gravidade do Trauma

## Abstract

**Introduction:**

The Revised Trauma Score (RTS) is one of the primary tools used to predict mortality in trauma patients. RTS is calculated at admission based on respiratory rate, systolic blood pressure, and Glasgow Coma Scale score. Its accuracy has been evaluated in several studies; however, most have predominantly included victims of blunt trauma, making its applicability to penetrating injuries a matter of ongoing debate.

**Objective:**

To compare the prognostic accuracy of the RTS for mortality prediction in patients with blunt trauma versus penetrating trauma.

**Methods:**

This retrospective cohort study included trauma patients treated at a referral hospital. The prognostic accuracy of the RTS for each trauma mechanism was assessed using receiver operating characteristic curves, and comparisons were performed using the DeLong test. Logistic regression analysis was conducted to identify additional independent predictors of trauma mortality. The chi-square test was used to determine a cutoff point for the RTS in penetrating trauma that would correspond to the reference cutoff adopted for blunt trauma.

**Results:**

A total of 3,575 patients were included. Of these, 2,448 (68.5%) sustained blunt trauma and 1,127 (31.5%) penetrating trauma. The area under the curve for mortality prediction was 0.813 in the blunt trauma group and 0.800 in the penetrating trauma group, with no statistically significant difference between them. Independent predictors of mortality included age, RTS value, acidosis, coagulopathy, and penetrating mechanism.

**Conclusion:**

The RTS is an accurate tool for predicting mortality in both blunt trauma and penetrating trauma.

## RESUMO

**Introdução:**

O Revised Trauma Score (RTS) é amplamente utilizado como ferramenta prognóstica no trauma, porém sua acurácia em lesões penetrantes permanece controversa. Considerando que esse mecanismo representa parcela significativa dos atendimentos no Brasil, torna-se essencial avaliar o desempenho do escore nessas populações.

**Objetivo:**

Comparar a acurácia prognóstica do RTS para predição de mortalidade entre vítimas de trauma contuso e penetrante, além de identificar um ponto de corte equivalente para indicação de transferência a centros de Trauma nível I.

**Materiais e Métodos:**

Trata-se de um estudo de coorte retrospectiva com pacientes atendidos entre 2015 e 2019 em um hospital de referência em Trauma. Foram analisados dados clínicos, laboratoriais e fisiológicos, com cálculo do RTS. A acurácia foi avaliada por curvas ROC e comparada pelo teste de DeLong. Regressão logística identificou preditores independentes de mortalidade.

**Resultados:**

Foram incluídos 3575 pacientes, 68,5% vítimas de trauma contuso e 31,5% de penetrante. A AUC do RTS foi de 0,813 para trauma contuso e 0,800 para penetrante (p=0,67). Lesões penetrantes aumentaram em 61% o risco de mortalidade. A equivalência entre mecanismos mostrou que um RTS ≤4,99 no trauma contuso corresponde a um RTS ≤6,99 no penetrante.

**Conclusão:**

O RTS apresenta acurácia semelhante para ambos os mecanismos de trauma. Entretanto, vítimas de trauma penetrante apresentam maior mortalidade, sugerindo a necessidade de ponto de corte mais elevado para triagem e transferência.

## INTRODUCTION

Defining a patient’s prognosis involves estimating the probability of developing a specific outcome in the future^1^ ^,^ ^2^. In trauma care, this is highly relevant for both prehospital care, when decisions are made regarding the most appropriate referral center, and hospital quality control, where comparisons are made between expected and observed clinical outcomes^1^ ^-^ ^4^. The Revised Trauma Score (RTS), one of the main prognostic tools in trauma care, evaluates and weights three parameters at admission: the Glasgow Coma Scale (GCS), respiratory rate (RR), and systolic blood pressure (SBP)^5^ ^-^ ^8^.

Several studies have demonstrated the accuracy of the RTS in predicting mortality^9^ ^-^ ^14^. However, most of these studies have focused on blunt trauma; in contrast, investigations including a higher proportion of penetrating injuries have reported conflicting findings. Studies such as those by Sacco et al.^15^ and Estumano et al.^16^ suggest that the RTS is a useful tool for prognosis in both types of injury. However, these results contrast with those reported by Alvarez et al.^3^ and Loh et al.^17^.

This inconsistency raises questions about the effectiveness of RTS in penetrating trauma, which accounts for approximately 20%-30% of trauma-related emergency admissions in Brazil^18^ ^-^ ^20^. Therefore, the aim of this study is to compare the prognostic accuracy of RTS as a mortality prediction tool in patients with blunt trauma and penetrating trauma, and to assess the practical equivalence of the score between these two populations.

## METHODOLOGY

### Study design and ethical approval

This retrospective cohort study was conducted in the emergency department of a trauma referral hospital in Brazil between July 2015 and December 2019. The protocol was submitted to the Human Research Ethics Committee at Instituto Gonçalo Moniz, which is affiliated with Fundação Oswaldo Cruz, and was approved on November 19, 2019, under opinion no. 3,712,006 and CAEE no. 21181519.1.0000.0040. Because of the retrospective nature of data collection, informed consent was waived.

### Sample selection

Patients were eligible for the study if they were admitted to the trauma service. Inclusion criteria were arrival alive at admission, followed by either hospitalization or death in the emergency department. Patients were excluded if they met at least one of the following criteria: i) being under 18 or over 60 years of age; or ii) having missing admission values for GCS, RR and/or SBP in their medical records. As all patients meeting the selection criteria were included in the study, a sample size calculation was not required.

### Variables

The protocol evaluated i) sociodemographic variables, including age, sex, type of trauma, and mechanism of injury; ii) vital signs at admission, including heart rate, RR, SBP, GCS, and SatO2; and iii) clinical variables, including injury topography, length of hospital stay, need for surgical intervention, admission to the intensive care unit (ICU), acidosis (defined as blood pH <7.35), coagulopathy (defined as international normalized ratio ≥1.5, platelet count <100,000, or partial thromboplastin time >40 seconds), and shock (defined as SBP <90 mmHg or diastolic blood pressure <60 mmHg, associated with at least one sign of tissue hypoperfusion: tachycardia > 120 bpm, GCS <12, capillary refill time ≥3 seconds, hemoglobin <12g/dL, arterial lactate > 2mmol/L, or base deficit <-2 mmol/L).

Laboratory test results were considered only if requested during the initial assessment and performed within 24 hours of admission.

### RTS Formula

The RTS was calculated using the formula below, where “v” represents a variable score ranging from 0 to 4 depending on the value of each included parameter, as detailed in Supplementary Material:

RTS=(0.9368×GCSv)+(0.7326×SBPv)+(0.2908×RRv)

Thus, the RTS ranges from 0 to 7.84 points, with higher values indicating a better prognosis. In this study, an RTS of ≤4.99 was adopted as the cutoff point for referral to a Level I trauma center^7^.

[Table t1a]

**Supplement 1 t1a:** “v” values for each parameter of the Revised Trauma Score (RTS).

GCS (score)	SBP (mmHg)	RR (breaths/min)	v
13 - 15	> 89	10 - 29	4
9 - 12	76 - 89	> 29	3
6 - 8	50 - 75	6 - 9	2
4 - 5	1 - 49	1 - 5	1
3	0	0	0

Abbreviations: GCS - Glasgow Coma Scale / SBP - Systolic Blood Pressure / RR - Respiratory Rate.

### Statistical analysis

Categorical variables were expressed as absolute values and percentages, while continuous variables were presented as median and interquartile range, according to the normality pattern identified by the Kolmogorov-Smirnov test. The Mann-Whitney U test was used to assess differences between groups for continuous variables, and the chi-square test or Fisher’s exact test for categorical variables.

The prognostic accuracy of the RTS for predicting mortality was evaluated using receiver operating characteristic (ROC) curves. One curve was generated for blunt trauma and another for penetrating trauma. They were then compared using the DeLong test. Logistic regression analysis was then performed to identify independent predictors of mortality within the study population.

Additionally, as the recommendation of RTS ≤4.99 as an indication for transfer to a Level I trauma center is primarily based on blunt trauma, we used the chi-square test to identify an equivalent cutoff point for penetrating trauma. We adopted mortality as the primary outcome and the need for surgery, ICU admission, and activation of the red wave protocol as secondary outcomes.

All statistical analyses were performed using the SPSS Statistics for Windows, version 14.0 (SPSS Inc., Chicago, Ill., USA). Statistical significance was set at p<0.05.

## RESULTS

### Characteristics of the sample

**Figure 1:**
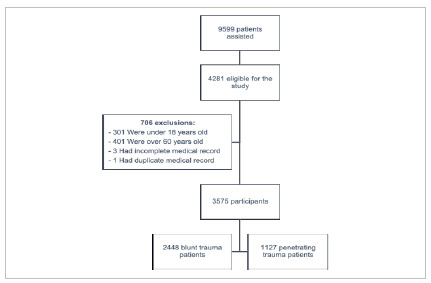
Flowchard of enrollment.

Between July 2015 and December 2019, 9,599 patients were admitted to the trauma department, of whom 4,281 were eligible for the study. Following the exclusion of 706 patients, the final sample comprised 3,575 individuals: 2,448 (68.5%) with blunt trauma and 1,127 (31.5%) with penetrating trauma (Figure 1).

In the blunt trauma group, the most common causes were motorcycle accidents (43.4%) and falls (21%). Injuries were most frequently located in the extremities (58.7%) and the head (53.6%). Of the patients in the penetrating trauma group, 857 (76%) sustained firearm injuries, most commonly to the extremities (49.2%), abdomen (44.1%) and chest (42.1%).

Mortality rates and the individual RTS components (GCS, RR, and SBP) differed significantly between the two groups. However, the overall RTS value did not differ between blunt and penetrating trauma (Table 1).

**Table 1:** Sample characteristics comparing all participants and non-survivors.

	Blunt Trauma (N = 2448)	Penetrating Trauma (N = 1127)	p-value	Blunt Trauma Non-survivors (N = 177)	Penetrating Trauma Non-survivors (N = 139)	p-value
Age - yr*	35 (27-45)	26 (22-34)	0.000	40 (30-52)	26 (22-34)	0.000
Male sex - no. (%)**	2077 (84.8)	1036 (91.9)	0.000	157 (88.7)	126 (90.6)	0.574
Mechanism of Trauma - no. (%)**			0.000			0.000
Gunshot wound	-	857 (76.0)		-	115 (82.7)	
Stab wound	-	246 (21.8)		-	22 (15.8)	
Fall	515 (21.0)	3 (0.3)		40 (22.6)	-	
Motorcycle crash	1063 (43.4)	6 (0.5)		48 (27.1)	1 (0.7)	
Car crash	223 (9.1)	1 (0.1)		20 (11.3)	1 (0.7)	
Bicycle	34 (1.4)	1 (0.1)		2 (1.1)	-	
Running over	280 (11.4)	1 (0.1)		36 (20.3)	-	
Agression	256 (10.5)	4 (0.4)		21 (11.9)	-	
Other	77 (3.1)	8 (0.7)		10 (5.6)	-	
	Blunt Trauma (N = 2448)	Penetrating Trauma (N = 1127)	p-value	Blunt Trauma Non-survivors (N = 177)	Penetrating Trauma Non-survivors (N = 139)	p-value
Lesion Topography - no. (%)**						
Head	1312 (53.6)	211 (18.7)	0.000	142 (80.2)	43 (30.9)	0.000
Cervical	174 (7.1)	120 (10.6)	0.000	10 (5.6)	17 (12.2)	0.038
Chest	356 (14.5)	475 (42.1)	0.000	48 (27.1)	70 (50.4)	0.000
Abdomen	431 (17.6)	497 (44.1)	0.000	36 (20.3)	77 (55.4)	0.000
Extremities	1438 (58.7)	555 (49.2)	0.000	59 (33.3)	49 (35.3)	0.721
Revised Trauma Score - score*	7.84 (7.84-7.84)	7.84 (7.84-7.84)	0.668	5.96 (4.09-7.32)	6.37 (4.09-7.84)	0.318
Glasgow Coma Scale - score*	15 (14-15)	15 (15-15)	0.000	6 (3-13)	12 (3-15)	0.001
Systolic Blood Pressure - mmHg*	128 (115-140)	120 (100-138)	0.000	120 (98.5-141.0)	90 (75-120)	0.000
Respiratory Rate - breaths/min*	20 (18-20)	20 (18-22)	0.000	20 (18-20)	20 (18-24)	0.147
Heart Rate - beats/min*	88 (78-100)	93 (80-110)	0.000	94 (76.5-120.0)	110 (81-124)	0.036
Shock - no. (%)**	117 (4.8)	197 (17.5)	0.000	34 (19.2)	72 (51.8)	0.000
Acidosis - no. (%)***	410/1574 (26.0)	387/936 (41.3)	0.000	95/162 (58.6)	90/124 (72.6)	0.018
Coagulopathy - no. (%)***	656/2162 (30.3)	467/1043 (44.8)	0.000	95/167 (94.4)	93/122 (76.2)	0.001
Polytrauma Classification - no. (%)***			0.000			0.000
Red wave	24/1875 (1.3)	174/828 (21.0)		13/141 (9.2)	58/94 (61.7)	
First route	590/1875 (31.5)	403/828 (48.7)		104/141 (73.8)	31/94 (33.0)	
Second route	1261/1875 (67.3)	251/828 (30.3)		24/141 (17.0)	5/94 (5.3)	
Hospital Stays - days*	6 (3-14)	4 (2-9)	0.000	5 (2-14)	1 (0-6)	0.000
Need of Surgery - no. (%)**	1729 (70.6)	978 (86.8)	0.000	93 (52.5)	115 (82.7)	0.000
Need of ICU - no. (%)**	613 (25.0)	289 (25.6)	0.7	128 (72.3)	69 (49.6)	0.000
Deaths - no. (%)**	177 (7.2)	139 (12.3)	0.000			

*Mann-Whitney U Test / **Chi-squared Test / ***Fisher’s Exact Test.

Among deceased patients, shock and acidosis were more prevalent in the penetrating trauma group, whereas coagulopathy was more common in the blunt trauma group. In addition, GCS values were significantly lower in patients with blunt trauma, while SBP values were lower in patients with penetrating trauma. However, no difference was observed in the overall RTS value between mechanisms (Table 1).

### RTS accuracy

**Figure 2:**
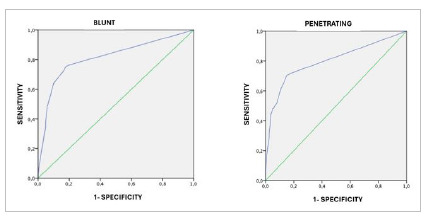
ROC curve for RTS mortality prediction.

The area under the ROC curve (AUC) for predicting mortality in cases of blunt trauma was 0.813 (standard error [SE], 0.02; 95% CI, 0.774-0.852; p=0.000), whereas in cases of penetrating trauma it was 0.800 (SE, 0.024; 95% CI, 0.752-0.847; p=0.000) (Figure 2). A comparison using the DeLong test showed no statistically significant difference between the two curves (p=0.67).

### Independent predictors

The logistic regression model was adjusted for the following factors: age; male sex; shock; acidosis; coagulopathy; RTS value; and penetrating mechanism. The final model (Table 2) demonstrated that, in addition to the RTS, penetrating injuries were independently associated with mortality, increasing the odds by around 61%.

**Table 2:** Logistic regression analysis and independent predictors of trauma-related mortality.

	B	SE	OR (95% CI)	p-value
Constant	0.382	0.417		
Age	0.028	0.007	1.02 (1.01 - 1.04)	0.000
RTS	-0.682	0.047	0.5 (0.46 - 0.55)	0.000
Acidosis	1.008	0.152	2.74 (2.03 - 3.69)	0.000
Coagulopathy	1.023	0.151	2.78 (2.06 - 3.74)	0.000
Penetrating injury	0.478	0.164	1.61 (1.17 - 2.22)	0.004

Abbreviations: SE - Standard Error / OR - Odds Ratio / CI - Confident Interval / RTS - Revised Trauma Score.

### Equivalence analysis

Patients in the blunt trauma group with an RTS ≤4.99 were compared with patients in the penetrating trauma group using different cutoff values. Mortality rates were equivalent between groups when an RTS ≤ 6.99 was used for penetrating trauma patients (Table 3).

**Table 3:** Equivalent cutoff point between blunt and penetrating trauma to indicate transfer to a level 1 trauma center.

	Blunt Trauma	Penetrating Trauma							
	RTS ≤4.99 (N = 176)	RTS ≤4.99 (N = 76)	p-value	RTS ≤5.99 (N = 113)	p-value	RTS ≤6 (N = 191)	p-value	RTS ≤7 (N = 1127)	p-value
Death - no. (%)	67 (38.1)	48 (63.2)	0.000	66 (58.4)	0.001	85 (44.5)	0.211	139 (12.3)	0.000
Need of Red Wave service - no. (%)	5 (2.8)	20 (26.3)	0.000	66 (58.4)	0.000	62 (32.5)	0.000	174 (15.4)	0.000
Need of Surgery - no. (%)	102 (58.0)	58 (76.3)	0.005	90 (79.6)	0.000	159 (83.2)	0.000	978 (86.8)	0.000
Need of ICU - no. (%)	147 (83.5)	42 (55.3)	0.000	62 (54.9)	0.000	98 (51.3)	0.000	289 (25.6)	0.000

## DISCUSSION

This study demonstrated that the RTS can predict mortality in trauma patients with approximately 80% accuracy, regardless of whether the injury is blunt or penetrating. However, other factors besides the RTS itself contribute to outcome prediction, including age, acidosis, coagulopathy and the penetrating mechanism. When considering mortality rates and the application of the score in the prehospital setting, an RTS ≤4.99 in victims of blunt trauma was found to be equivalent to an RTS ≤6.99 in patients with penetrating injuries.

The findings of this study are consistent with those reported by Kuhls et al.^21^ and Yousefzadeh-Chabok et al.^11^, both of whom compared different trauma mortality prediction scores and reported AUC values for the RTS greater than 0.8 (0.84 and 0.87, respectively). In contrast, Loh et al.^17^ reported an AUC of 0.618. A possible explanation for this discrepancy may lie in the study population. Loh et al.^17^ evaluated a specific sample of patients with vascular injuries, and given the progression of hypovolemia, the physiological parameters assessed by the RTS may only show marked alterations in the presence of class III shock^22^. This condition may not yet be established at the time of admission, when the score is calculated.

Additional findings from the present study support this interpretation. In the logistic regression analysis, shock was not retained as an independent predictor of mortality, whereas acidosis and coagulopathy were. This observation is indirectly supported by studies aimed at improving trauma scoring systems, such as those by Jeong et al.^23^ and Filipescu et al.^24^.

Moreover, our results showed that mortality was higher among patients with penetrating injuries compared with those with blunt trauma, which is consistent with findings reported by Alvarez et al.^3^ and Romeo et al.^25^ In seeking equivalence between the two mechanisms, we identified that, in penetrating trauma, an RTS ≤6.99 corresponds-regarding mortality rates-to the RTS ≤4.99 cutoff adopted for blunt trauma. The study by Loh et al.^17^ supports this interpretation, as the group with higher mortality in their cohort had a mean RTS of 6.64.

This study has some limitations. Its retrospective design makes primary data collection dependent on the interpretation and documentation of health professionals. However, this limitation was partially mitigated by the use of standardized electronic equipment for vital sign measurement at the hospital. In addition, the request for laboratory tests depended on individual physician decisions, which led to missing data; this issue was addressed through appropriate statistical handling. Finally, although our findings are consistent with those of other studies, this was a single-center investigation, and caution should be exercised when generalizing the results.

## CONCLUSION

The RTS is an accurate tool for predicting mortality in both trauma mechanisms, blunt and penetrating. Mortality among patients with penetrating injuries is significantly higher, and an RTS ≤6.99 in this group is statistically comparable, in terms of mortality prediction, to an RTS ≤4.99 among victims of blunt trauma. Further studies are needed to confirm this hypothesis.
